# The Impact of Telemedicine during the COVID-19 Pandemic on Diabetes Management and Pregnancy Outcomes in Women with Gestational Diabetes Mellitus (GDM)

**DOI:** 10.3390/jcm13195797

**Published:** 2024-09-28

**Authors:** Edyta Cichocka, Janusz Gumprecht

**Affiliations:** Department of Internal Medicine, Diabetology and Nephrology Faculty of Medical Sciences in Zabrze, Medical University of Silesia, 40-055 Katowice, Poland; jgumprecht@sum.edu.pl

**Keywords:** gestational diabetes mellitus, COVID-19 pandemic, telemedicine

## Abstract

**Background/Objectives**: Gestational diabetes mellitus (GDM) can lead to various complications for both the mother and the child. Many factors influence the onset of the disease including GDM in a previous pregnancy, overweight and obesity, as well as the increasing age of women who become pregnant. The aim of this study was to assess the impact of telemedicine during the COVID-19 pandemic on diabetes management and pregnancy outcomes in women with gestational diabetes mellitus (GDM). **Methods:** A retrospective analysis was conducted. We compared two groups of GDM women from the pandemic and pre-pandemic periods in terms of pregnancy course and obstetric outcomes. **Results:** It was observed that women with GDM in the pandemic group were significantly more often overweight and significantly less often obese before pregnancy than women in the pre-pandemic group. GDM was diagnosed significantly earlier but in later pregnancies in the pandemic group than in the pre-pandemic group. The course of pregnancy in both groups was similar and the percentage of pregnancies with the delivery of neonates > 4000 g did not differ significantly. The number of caesarean sections and spontaneous deliveries was also similar in both periods. No differences were found in anthropometric parameters in newborns and neonatal and maternal complications. **Conclusions:** The occurrence of the COVID-19 pandemic and the necessity of employing telemedicine tools in the medical care of women with GDM did not significantly impact diabetes management and the pregnancy outcomes achieved.

## 1. Introduction

Gestational diabetes mellitus (GDM), which is defined as a disorder of carbohydrate metabolism that occurs during pregnancy, is one of the most common metabolic complications during pregnancy [1]. Predisposition to GDM is present before pregnancy, and hormonal changes and weight gain during pregnancy finally lead to the manifestation of GDM [1,2]. GDM can lead to various complications for both the mother and the child, including an increased risk of hypertension, pre-eclampsia, premature birth, macrosomia, and stillbirth. It is also a strong prognostic factor for metabolic disorders in the child, both in childhood and adulthood [1,2].

Many factors also influence the onset of the disease, including GDM in a previous pregnancy, overweight and obesity, as well as the increasing age of women who become pregnant [1]. The COVID-19 pandemic should also be considered a significant environmental factor, as it was associated with a markedly higher level of stress and anxiety regarding one’s life and health, as well as with restrictions related to daily activities, including periods of lockdown, prolonged isolation at home, social distancing, and limited opportunities for exercise (the closure of sports clubs). This was also a time of remote work, promoting a sedentary lifestyle and challenging access to medical care. All of the above-mentioned factors contributed to a troubling increase in the incidence of diabetes, both type 1 and type 2, as well as GDM, during the COVID-19 pandemic [3].

According to the National Vital Statistics Reports of the United States, the incidence rate of GDM in 2020 was 30% higher compared to 2016 and amounted to 7.8 per 100 births [4].

A similar trend was also observed in Europe; in the northeastern region of Italy, which was heavily affected by the pandemic, Zanardo et al. reported that the COVID-19 pandemic had a negative impact on the incidence of gestational diabetes mellitus (GDM) in 2020 compared to 2019 [5]. In a retrospective study, Mirsky et al. found that the prevalence of GDM increased by 38.9% during the COVID-19 pandemic compared to pre-pandemic figures [6].

Due to significant lifestyle changes during the pandemic, including reduced opportunities for exercise, it is believed that the COVID-19 pandemic was associated with increased gestational weight gain (GWG) during pregnancy. A study conducted in Italy during the COVID-19 pandemic lockdown revealed that pregnant women had higher body mass indices (BMIs) and experienced increased weight gain during pregnancy [7].

The reduction in in-person visits to healthcare facilities to minimize the risk of infection during the pandemic particularly affected the management of women with gestational diabetes. Most medical facilities implemented telemedicine services during this time, which included telephone calls, video consultations, and the use of online platforms and mobile applications for managing and monitoring gestational diabetes [8].

The aim of this study was to assess the impact of telemedicine during the COVID-19 pandemic on diabetes management and pregnancy outcomes in women with gestational diabetes mellitus (GDM) treated in the Diabetes Outpatient Clinic of Clinical Hospital No. 1 in Zabrze, Poland.

## 2. Materials and Methods

We conducted a retrospective study. The medical records of GDM women treated during the COVID-19 pandemic in the Diabetes Outpatient Clinic in Zabrze were analyzed. All women were diagnosed with GDM on the basis of the current criteria (WHO 2013) and only single gestations were included in this study. The exclusion criteria included pregnancy in women with other types of diabetes and multiple pregnancy, as well as lack of consent to participate in this study. During the pandemic, after the diagnosis of GDM, women had their first visit in person, while subsequent visits (every 3–4 weeks on average) were conducted using telemedicine tools. On the first visit, women received diabetes education about glycemic targets and appropriate weight gain. Dietary training with a dietician, as well as guidance on self-monitoring, were carried out. The visits were more frequent when target glycemic values were higher for seven consecutive days (fasting or postprandial). Insulin therapy was initiated if glycemia exceeded target values for seven consecutive days. We included in the analysis only women who completed their pregnancies. Data were collected from medical records, with information about the delivery being obtained through telephone contact with the women. A total of 66 patients’ documentation from 2021 to 2022 was analyzed. The results were compared to a group of patients with GDM from before the pandemic—years 2018–2019—who were under the supervision of the Diabetes Clinic during their pregnancies (65 patients). Patients from the pre-pandemic period attended all their visits to the Outpatient Clinic in person. All of them were also trained in glucose self-monitoring (control of fasting glucose and glucose 1 h after the start of the meal) and monitoring ketone bodies in urine in the morning during the first visit. Dietary training related to the principles of proper nutrition was also conducted (with the recommendations of a balanced diet containing an average of 25–30 kcal/kg, depending on the initial body weight before pregnancy). Visits took place every 4 weeks on average. However, they were more frequent when (fasting or postprandial) target glycemic values were higher for 7 consecutive days. Insulin therapy was initiated if glycemic targets for self-monitoring were higher for 7 consecutive days. Each patient provided consent for the use of their medical data. A retrospective study did not require the consent of the Bioethics Committee.

### Statistical Analysis

The analysis was performed using the R language in RStudio (2024.04.2+764.pro1) To assess the normality of the variables, we used a histogram and a quantile–quantile (QQ) plot. Quantitative variables with a normal distribution were presented as means with standard deviation and variables with deviations from the normal distribution were presented as the median with the first and third quartiles. For statistical analyses of quantitative variables with a normal distribution, Student’s *t*-test was used. The Wilcoxon test was applied for quantitative variables with a deviation from the normal distribution. The relationships between the qualitative variables were assessed using the Pearson chi-square test. Correlations were assessed using Spearman’s rank correlation coefficient. *p*-values < 0.05 were considered statistically significant.

## 3. Results

The general characteristics of the study groups are presented in Table 1.

In this study, it was observed that patients in the COVID-19 group were significantly more often overweight and significantly less often obese before pregnancy than patients in the 2018/2019 group (Table 2). GDM occurred in an earlier week of pregnancy in the COVID-19 group than in the 2018/2019 group (18 weeks of pregnancy vs. 24 weeks of pregnancy, *p* = 0.032) (Table 1). In the COVID-19 group, GDM occurred in later pregnancies than in the 2018/2019 pregnant group. This relationship was statistically significant (*p* = 0.00617) (Table 1).

**Figure 1:**
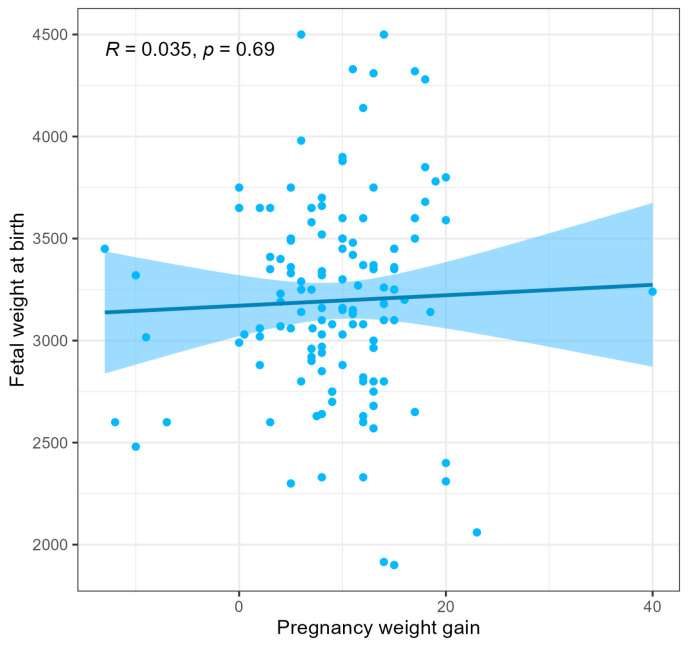
Correlation between pregnancy weight gain and fetal weight at birth. 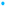 dispersion of patients on the x and y axes.

**Figure 2:**
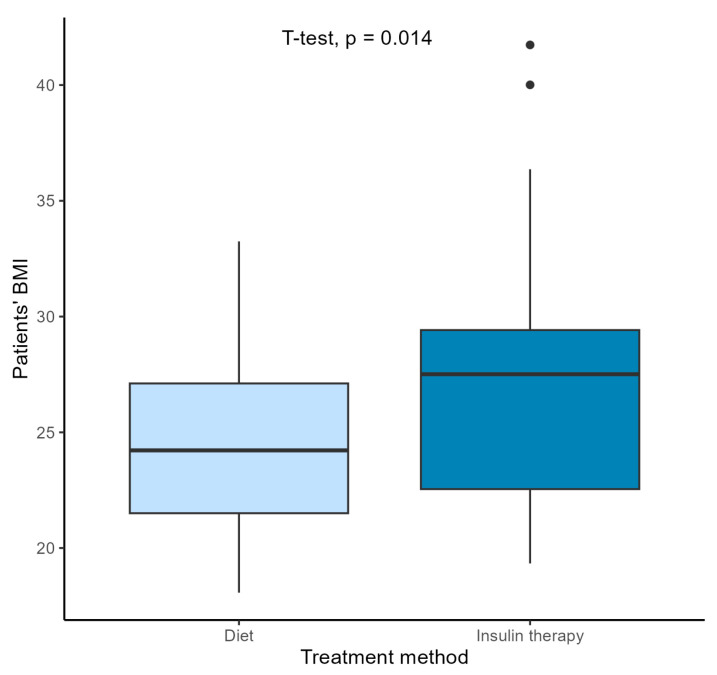
Comparison of patients’ BMI by treatment method (Student’s *t*-test); 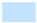 diet treated patients; 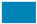 insulin treated patients.

**Figure 3:**
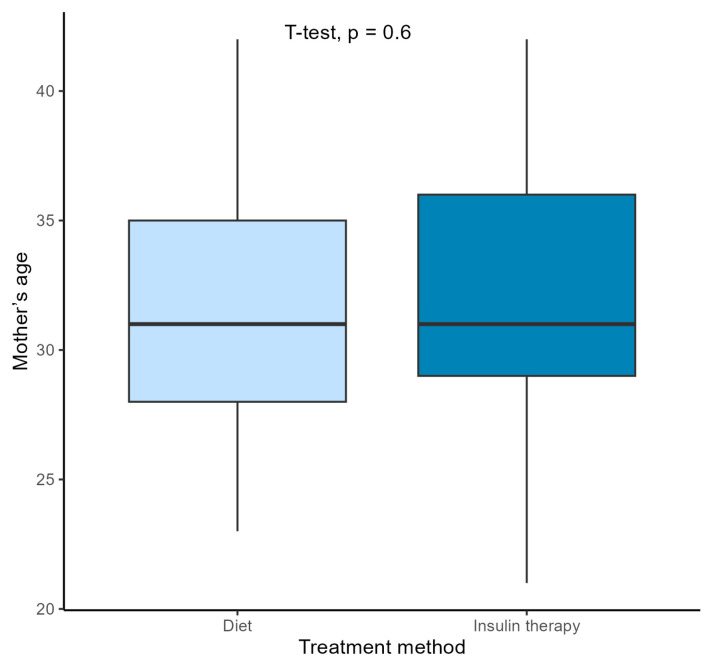
Comparison of the treatment methods and mother’s age (Student’s *t*-test); 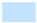 diet treated patients; 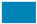 insulin treated patients.

No significant differences were found in terms of the other assessed parameters between the groups of pregnant women from the 2018/2019 and the COVID-19 groups. In both groups, women had similar ages at conception. The week of delivery was also similar. The number of cesarean sections and spontaneous deliveries was similar in both periods (Table 2). In both groups, insulin treatment was started at a similar stage of pregnancy. There were also no differences between the parameters in newborns in terms of birth weight, body length, or Apgar scores at 1 min. During pregnancy, patients achieved similar weight gain (averaging 9.24 kg and 9.59 kg, respectively). However, when analyzing weight gain based on the treatment method (Table 3), it was shown that patients in the 2018/2019 group who were treated with insulin gained weight significantly less compared to those treated with dietary training only (*p* = 0.039). In the COVID-19 group, this relationship was not observed (*p* = 0.059). Nevertheless, this did not translate into the newborns’ weight at birth. A comparison of newborns with a birth weight > 4000 g showed no differences among the groups—6% of infants in both groups were born with a birth weight > 4000 g (Table 2). No significant correlation was observed between maternal weight gain during pregnancy and fetal weight at birth (Figure 1). GDM patients treated with insulin therapy had significantly higher BMIs before pregnancy than those treated with dietary training (Figure 2). No difference was observed in the treatment of patients based on their age (Figure 3).

Maternal comorbidities were compared between the groups. No difference was found in the prevalence of hypothyroidism, arterial hypertension, or polycystic ovary syndrome (PCOS) (Table 4).

Neonatal complications were also assessed. No difference was found in the prevalence of neonatal hypoglycemia which required medical attention, prolonged jaundice, and heart defects (Table 5).

In the COVID-19 group, 50% of patients had suffered from COVID-19 and 59% had been vaccinated (only patients who completed a series of vaccinations with two doses (Pfizer/BioN Tech) or one dose (Johnson & Johnson) were included in the analysis). Over 50% of pregnant women did not think that the pandemic had a negative impact on their pregnancies and, interestingly, over 60% of them preferred a visit using telemedicine tools instead of an in-person visit (Table 6).

## 4. Discussion

The COVID-19 pandemic raised serious concerns regarding the diagnosis and treatment of pregnant women and complicated routine prenatal care by limiting access to in-person visits as well as laboratory testing [9]. These changes also affected the treatment and diagnosis of GDM. The performance of the OGTT became a challenge for healthcare providers, leading some countries to abandon it and instead incorporate HbA1c, fasting plasma glucose (FPG), and random plasma glucose (RPG) into the diagnostic criteria (Canada, Australia, and New Zealand) [10,11]. However, it should be noted that the available evidence supporting the use of RPG, HbA1c, and FPG in diagnosing GDM is relatively limited compared to the use of the “gold standard”, which is the OGTT. In Poland, no changes were made regarding GDM diagnosis during the COVID-19 pandemic, and all patients routinely underwent the OGTT.

During the pandemic, it was necessary to reorganize and adapt the healthcare system to ensure proper care and treatment for pregnant women. The utilization of telemedicine, including phone calls, online platforms, and mobile applications, played a crucial role in facilitating the diagnosis, treatment, and control of GDM [8], as well as reducing the risk of exposure to COVID-19.

A study conducted by Munda et al. [12] confirmed, similar to our study, that transitioning to remote visits for patients with GDM does not worsen glycemic control or lead to adverse outcomes for newborns. Kozica-Olenski et al. [13], in their observation, pointed out the difficulties related to the quality of the Internet, sound, and visuals that disrupted remote consultations. Problems also arose in receiving prescriptions for insulin and insufficient access to dietary and lifestyle counseling. These barriers were particularly evident among individuals who were not fluent in the language used during consultations. Additionally, more than half of the women surveyed in the study conducted by Kozica-Olenski et al. [13] did not feel confident about self-monitoring their weight and blood pressure. Overall, women expressed satisfaction with phone consultations, as they provided access to high-quality care during the pandemic. However, a significant majority clearly preferred in-person care and believed that telemedicine deteriorated the quality of their healthcare [13,14].

In our presented study, over 60% of patients preferred a phone appointment, and 54.5% did not believe that the pandemic negatively affected the quality of healthcare during their pregnancy. Furthermore, the obstetric outcomes obtained, which did not differ significantly from those achieved before the pandemic, indicate a high standard of care in our country.

As previously described, in our center, nearly all pregnant women diagnosed with GDM had their first visit conducted in person (95.5%), during which they received education on diabetes management and diet, which may explain their satisfaction with subsequent visits utilizing telemedicine tools. It is worth noting that patients requiring insulin therapy for GDM before the pandemic had better weight control and gained less compared to those treated with insulin during the pandemic. In-person visits may have allowed for better monitoring of weight gain, especially in the group of patients treated with insulin.

Similar results to those presented above were obtained in another diabetes center in Poland. Wilk M et al. assessed the impact of the first wave of the COVID-19 pandemic on the quality of care for women with GDM. The authors reported that the first wave of the COVID-19 pandemic seemed to not have caused a negative impact on glycemic control and pregnancy outcomes in GDM women, in spite of reported difficulties in diabetes management delivery [15].

The authors are aware that a limitation of this study is the relatively small groups of patients being compared, and the results, especially regarding neonatal complications, should be interpreted with great caution. It should also be emphasized that patients with GDM are most often under the supervision of an outpatient diabetes clinic only until delivery, and it is not always possible to obtain complete data in terms of delivery or neonatal complications at a later stage.

It is important to remember that a history of previous GDM, higher pre-pregnancy body weight, or advanced maternal age warrants active screening for GDM in the early stages of subsequent pregnancy. Patients should be informed about the risk of developing type 2 diabetes later in life. Emphasizing education on proper diet, physical activity, and the importance of maintaining a healthy body weight is crucial in reducing this risk.

## 5. Conclusions

The occurrence of the COVID-19 pandemic and the necessity of employing telemedicine tools in the medical care of women with GDM did not significantly impact diabetes management and pregnancy outcomes achieved in this study.

Regardless of the method of care for pregnant women with GDM, whether in person or utilizing telemedicine tools, it should be remembered that a pregnant woman requires a multifactorial approach involving self-monitoring (including continuous glucose monitoring systems (CGMs)), maternal monitoring of weight gain, and fetal growth assessment.

Our study adds further evidence that the healthcare system in Poland managed this challenging period effectively. The combined efforts of healthcare professionals, the motivation of our patients to cooperate, and the effective use of telemedicine tools allowed these women to deliver similarly healthy newborns during the pandemic as before.

This study may have practical implications beyond the pandemic period, when we will safely use telemedicine tools to sufficiently treat diabetic persons not only with GDM but also with other types of diabetes, thereby achieving good diabetes management.

## Figures and Tables

**Table 1:** Characteristics of the study groups.

Variable	GDM in the 2018/2019 Group (n = 65)			GDM in the COVID-19 Group (n = 66)		*p*	
	Mean	SD		Mean	SD		
Age, years	30.9	4.56		32	4.6	0.187	
Body weight before pregnancy, kg	73.2	21.4		68.9	13.4	0.294	
BMI, kg/m^2^	26.3	6.4		25.3	4	0.47	
Body weight gain during pregnancy, kg	9.24	8.06		9.59	5.83	0.776	
Week in which GDM was diagnosed	24	7		18	9	<0.001	
Week of delivery	38.1	2.12		38.4	1.61	0.43	
Neonatal body weight at delivery, g	3220.0	531		3180.0	503	0.635	
Neonatal body length at delivery, cm	53.7	3.18		53.7	3.06	0.937	
	**Median**	**Q1**	**Q3**	**Median**	**Q1**	**Q3**	***p***
Week in which insulin was started	26.5	19.5	30.2	28	14	32	0.690
Order of pregnancy in which GDM occurred	1	1	2	2	1	3	0.00617
Apgar score at 1 min	10.00	9.00	10.00	10.00	10.000	10.00	0.0855

BMI—body mass index [kg/m^2^], GDM—gestational diabetes mellitus.

**Table 2:** Comparison between other parameters in both groups.

Variable	18/19 Group, n = 65	COVID-19 Group, n = 66	*p*
BMI before pregnancy			0.032
<25	59%	45%	
≥25	15%	43%	
≥30	26%	12%	
Order of pregnancy in which			0.060
GDM occurred			
1	58%	38%	
2	24%	31%	
3	8%	25%	
4	8%	2%	
5	2%	2%	
6	0%	2%	
Diagnosis of GDM (OGTT)			0.33
Fasting	22 (34%)	25 (38%)	
2 PG	19 (29%)	11 (17%)	
1h PG, 2h PG	7 (11%)	8 (12%)	
1h PG	7 (11%)	6 (9%)	
Fasting, 1h PG, 2h PG	3 (5%)	10 (15%)	
Fasting, 1h PG	4 (6%)	2 (3%)	
Fasting, 2h PG	3 (5%)	3 (5%)	
Mother over the age of 35	55 (85%)	48 (73%)	0.15
Caesarean sections/spontaneous delivery	52%/48%	57%/43%	0.72
Neonatal body mass at delivery:			>0.99
<4000 g	94%	94%	
>4000 g	6%	6%	

BMI—body mass index, OGTT—oral glucose tolerance test, PG—plasma glucose.

**Table 3:** Body weight gain during pregnancy according to the mode of treatment of GDM.

Variable		n	Median	Q1	Q3	*p*
GDM in the 2018/2019 group	diet	42	10	7	15	
	insulin	23	7.5	2.25	11.8	*p* = 0.039
GDM in the COVID-19 group	diet	35	11.5	8	13	
	insulin	31	9	6	12	*p* = 0.059

**Table 4:** Maternal comorbidities.

Maternal Comorbidities	2018/2019 GDM Group (*n* = 65)		COVID-19 GDM Group (*n* = 66)		Chi^2^ Test
Arterial hypertension	6	4.58%	7	5.344%	*p* = 1
Hypothyroidism	18	13.74%	28	21.374%	*p* = 0.1134
Polycystic ovary syndrome	10	7.64%	6	4.58%	*p* = 0.4048

**Table 5:** Neonatal comorbidities.

Neonatal Comorbidities	18/19 GDM Group (*n* = 65)		COVID-19 GDM Group (*n* = 66)		Chi^2^ Test
Hypoglycemia	3	2.29%	1	0.763%	*p* = 0.6007
Prolonged jaundice	10	7.63%	6	4.58%	*p* = 0.4048
Heart defects	2	1.527%	0	0%	*p* = 0.4694

**Table 6:** Influence of COVID-19 on pregnancy.

	COVID-19 GDM Group (*n* = 66)	
Type of first visit	In-person	63 (95.5%)
	Teleconsultation	3 (4.5%)
What kind of visit do you prefer?	In-person	9 (13.6%)
	Teleconsultation	40 (60.6%)
	Both	7 (10.6%)
	I do not know	10 (15.2%)
Past COVID-19 infection?	No	33 (50%)
	Yes	33 (50%)
COVID-19 vaccination?	No	27 (41%)
	Yes	39 (59%)
Did the pandemic negatively affect your pregnancy?	No	36 (54.5%)
	Yes	30 (45.5%)

## Data Availability

Data are contained within the article.
